# Obesity and Atrial Fibrillation: Should We Screen for Atrial Fibrillation in Obese Individuals? A Comprehensive Review

**DOI:** 10.7759/cureus.10471

**Published:** 2020-09-15

**Authors:** Kyrillos N Ghattas, Shahbakht Ilyas, Reham Al-Refai, Reeju Maharjan, Liliana Diaz Bustamante, Safeera Khan

**Affiliations:** 1 Internal Medicine, California Institute of Behavioral Neurosciences and Psychology, Fairfield, USA; 2 Surgery, California Institute of Behavioral Neurosciences and Psychology, Fairfield, USA; 3 Pathology, California Institute of Behavioral Neurosciences and Psychology, Fairfield, USA; 4 Neurology, California Institute of Behavioral Neurosciences and Psychology, Fairfield, USA; 5 Family Medicine, California Institute of Behavioral Neurosciences and Psychology, Fairfield, USA

**Keywords:** obesity, atrial fibrillation, left atrium remodeling, epicardial adipose tissue, obesity-related illnesses

## Abstract

Obesity and obesity-related illnesses (ORIs) constitute a significant burden on the healthcare system, with a very high prevalence in the general population. Atrial fibrillation (AF) is the most common arrhythmia seen by healthcare providers. The risk of AF in obese individuals is reported to be high and in correlation with Body Mass Index (BMI), leading to the high prevalence of AF in the general population and the expected epidemic of AF to come. Greater left atrial dimensions and left atrial remodeling together form the AF substrate in the obese population along with the role of epicardial adipose tissue (EAT) in inducing inflammation and fibrosis of the atrial myocardium and thus facilitating the onset of AF. In our paper, we reviewed the literature published on the link between obesity and AF, as well as the potential behind new management approaches. Multiple studies have explored different approaches, either conventional or novel. Considering the impact of prevention in medicine nowadays, we proposed a screening practice for AF in obese individuals. More research is needed to acquire a comprehensive protocol for the management of AF in the obese population that can be applied by primary healthcare providers to combat this evolving matter.

## Introduction and background

Obesity is unceasingly on the rise worldwide, accompanied by obesity-related illnesses (ORIs). ORIs include, but are not limited to, cardiovascular diseases (CVDs), diabetes mellitus (DM), musculoskeletal disorders especially osteoarthritis, and some cancers including endometrial, breast, ovarian, and prostate according to World Health Organization (WHO) fact sheet on obesity. The prevalence of obesity has doubled in more than 70 countries worldwide since 1980 and there were 603.7 million obese adults in 2015 [1]. In the USA, the prevalence of obesity in adults was about 39.8% in 2015-2016 [2]. Unsurprisingly, obesity places a major stress on the cardiovascular system, contributing to many CVDs, including atrial fibrillation (AF) [3]. AF is the most common arrhythmia in clinical practice with experts predicting its prevalence to increase three-fold in the next three decades, classifying AF as a potential epidemic that all healthcare providers should be aware of its management [4]. Provided that medical researchers are obliged to look for improved protocols for diagnosis and treatment of both obesity and AF.

Many studies have already explored the clinical link between obesity and AF, identifying obesity as a risk factor for the development of AF. One study found that obesity represents a novel risk factor for AF associated with an increase in the incidence of 50% [5]. One of the most common triggers for AF is micro-reentry and enhanced automaticity in one or more of the circuits of the atria [6]. Obesity can contribute to the pathophysiology of AF in many direct and indirect mechanisms, including cardiometabolic abnormalities, mechanical effects, inflammation, and increased adiposity, all leading to left atrium (LA) remodeling, which is a known substrate for AF [7].

A few recent studies have focused on the pathophysiologic aspect of the relationship between obesity and AF. One review article points towards accumulating evidence that this relationship is not an epiphenomenon but rather the result of complex crosstalk between the epicardial adipose tissue (EAT) and the adjacent myocardium through the effect of adipokines, inflammatory cytokines, or reactive oxidative species [8]. Another article suggests that common pathophysiological pathways between obesity and certain conditions such as hypertension, DM, metabolic syndrome, obstructive sleep apnea (OSA), and coronary artery disease (CAD) could potentially be functioning and contributing to the progression of AF in such patients [9].

In this review, we analytically examine existing evidence on AF risk in the population of obese patients with or without ORIs. Certain characteristics will be discussed concerning this population: a) incidence and prevalence of AF; b) pathophysiological aspects; c) potential approaches to management and treatment of AF. Taken together, these innovative acumens point to the development of new screening strategies to battle the weight, progression, and healthcare cost of AF in patients with obesity.

## Review

Incidence and prevalence of AF in the obese population

To have more efficient healthcare systems, more research is needed on the level of prevention and early detection of clinical disorders. Understanding the prevalence of AF in the obese population will tremendously help early detection and will play a major role in toning down the forthcoming epidemic of AF. Research looking into the pathophysiology of AF in the obese population will help to achieve prevention on a long-term basis. Foy et al. noticed that obese individuals were more frequently diagnosed with hypertension and DM, which suggests a pathophysiologic contribution to new-onset AF. The difference in the incidence of AF after one year was 0.1 compared to after eight years which was 0.9 suggesting the role of aging in the development of new-onset AF. However, odds ratio (OR) was 1.4, 95% Confidence Interval (CI) 1.26-1.56 in obese patients after controlling for age, as well as hypertension, DM, and gender [10].

In a cross-sectional study, results showed a prevalence of 16% for AF and 33% for obesity in a total of 499 patients [11]. However, it is important to mention that the initial cohort of patients recruited for the study was patients who underwent transthoracic echocardiography at an academic tertiary care teaching hospital. Speculations of incidence and prevalence of AF in the general population were of close numbers in the meta-analysis by Wanahita et al., where they concluded that AF risk is drastically higher in obese individuals [12]. A retrospective cohort study in 2005 investigated the incidence of AF in obese patients following cardiac surgery. The study included 8051 patients, and the results showed an association between Body Mass Index (BMI) and new-onset AF, where the risk for AF increases by 1% for every one-unit increase in BMI [13]. Using the Framingham Heart Study and the Framingham Offspring Study, Wang et al. in their cohort study concluded that obesity is a substantial risk factor for AF. After almost 14 years of follow-up, the incidence of AF after adjusting for age increased in line with all categories of BMI in men and women. An analysis adjusted for cardiovascular risk factors and cardiac pathologies, for the same study, obesity remained a significant risk factor for AF as each unit increase of BMI was associated with a 4% increase of AF risk in both men and women [5]. Table 1 shows a comparison between all five studies.

**Table 1:** A comparison table between all five studies AF = Atrial Fibrillation, DM = Diabetes Mellitus, OR = Odds Ratio, LA = Left Atrium, BMI = Body Mass Index

Author and Year of Publication	Intervention	Type of Study and Number of Patients	Results	Conclusion
Foy AJ et al., 2018 [10]	Review of patients’ data from 1/1/2006 to 12/31/2013.	Prospective Cohort Study, 67,278 patients	Obesity is strongly associated with new-onset AF after controlling for age, gender, hypertension, and DM (OR 1.4, 95% CI1.26 – 1.56).	The study finds evidence supporting the causal relationship between obesity and AF and emphasizes the need to identify obesity as a measure of management to prevent/treat AF.
Huang G et al., 2017 [11]	Review of patients’ data between May 2010 and June 2010.	An observational study, 499 patients	Obese patients had larger LA diameters, LA areas, LA volumes than normal-weight patients. BMI is independently associated with LA diameter, LA area, and LA volume in multivariate analysis.	The prevalence of AF and obesity in the overall population were 16% and 33%, respectively. Higher BMI is associated independently with increased LA diameter, LA area, and LA volume.
Wanahita N et al., 2008 [12]	To evaluate obesity as a risk factor for the development of AF	Meta-analysis, 123,249 patients	Obese patients have an increased risk of 49% to develop AF compared to nonobese patients.	AF risk is augmented by 49% in the obese population compared to the general population, and the risk increases hand in hand with increased BMI.
Zacharias A et al., 2005 [13]	A retrospective study of the incidence of AF concerning BMI after cardiac surgery.	Retrospective Cohort Study, 8051 patients	BMI was significantly associated with AF with a 1% increase in AF incidence with each unit increase in BMI.	The data show that the higher the obesity rate the more the chances of developing new-onset AF after cardiac surgery.
Wang TJ et al., 2004 [5]	To examine the association between BMI and the risk of developing AF.	An observational study, 5282 patients	In multivariable models, each unit increase of BMI was associated with a 4% increase of AF risk in both men and women.	Obesity is a risk factor for AF. The excess risk of AF associated with obesity is thought to be facilitated by LA dilatation.

Pathophysiological aspects of AF in the obese population

Mechanisms of AF in obesity can be complex and overlapping. Research has identified multiple molecular and cellular pathophysiological determinants that may lead to changes in the atria and thus favoring the occurrence of AF. In our article, we identified two evidence-based major mechanisms that can be attributed to the development of AF in an obese population: a) LA dimensions; and b) The role of EAT and LA remodeling. 

LA Dimensions

Notably, LA enlargement is demonstrated to be present in the obese population even if they are free of any cardiovascular conditions, and such measurements can be noticeable on echocardiography without markers of diastolic dysfunction [14]. In a study on a specific subgroup of AF patients who were diagnosed with persistent AF right from the onset to determine the clinical characteristics and prognosis of such group, Lim et al. established interesting results regarding certain demographics. The study recruited 360 patients who were referred for persistent AF ablation, of whom 129 patients were diagnosed with persistent AF from the onset, and 231 patients had progressed from paroxysmal to persistent AF. Results showed that patients with persistent AF from the onset were statistically significantly more obese and, more interestingly, had larger dimensions of both the left and right atria. After follow-up for a range of time between six and 28 months after the ablation, patients with persistent AF from the onset had higher AF recurrence rates and generally worse prognosis. In the same study, 90 patients were recruited for noninvasive mapping of AF drivers as well as invasively assessing the degree of fractionation and endocardial voltage. Recruited patients were divided into two groups: 30 patients with persistent AF from the onset and 60 control individuals. Results demonstrated a significantly shorter baseline AF cycle length in patients with persistent AF from the onset vs. the control group. Additionally, patients with persistent AF from the onset showed lower endocardial voltage than the control group in both atria [15].

Hypertension can play a significant role in LA remodeling and in distorting LA volume. A study found that patients with obesity have more risk of increased LA volume than hypertensive patients. Additionally, after categorizing the studied population, a normal-weight hypertensive subgroup of patients was found to have significantly lower LA volumes than obese normotensive individuals, also, unsurprisingly, the subgroup of the obese hypertensive population had the highest LA volumes [16]. Tsang et al. in their cohort study in Olmsted County, MN concluded that there is more than a 150% increase in risk of progression of paroxysmal AF to permanent AF in population with BMI ≥30 kg/m2 along with a graded risk relationship between BMI and progression of paroxysmal to permanent AF, in which case the risk was augmented by larger LA dimensions [17]. BMI is a significant predictor of LA size [18]. Size and dimensions are pivotal concerning the pathologies of the atria. LA dilation leads to significant remodeling as well as changes in molecular and physiologic levels.

The main characters of metabolic syndrome are elevated blood pressure, abdominal obesity, insulin resistance, and dyslipidemia (high triglycerides and low high-density lipoprotein cholesterol) [19]. Metabolic syndrome is mostly a natural consequence of long-term untreated obesity and potentially could lie under the umbrella of ORIs. It has been demonstrated that there is a significant association between metabolic syndrome and atrial size in patients with nonvalvular paroxysmal AF [20]. Huxley et al. found a significant relationship between DM, as one of the ORIs, and incident of AF with increased AF risk proportional to poor glycemic control [21].

The Role of EAT and LA Remodeling

The volume of EAT measured by magnetic resonance imaging (MRI) is statistically associated with AF independent from other known risk factors of AF. It has been shown that patients with persistent AF have higher EAT volumes than patients with paroxysmal AF [22]. In the Framingham Heart cohort study, pericardial fat volume measured by computed tomography (CT) appears to be a predictor of AF as well as BMI [23]. There is growing evidence of the role of inflammation in AF and experimental data shows that activation of the renin-angiotensin-aldosterone system (RAAS) plays a crucial role in the process through multiple inflammatory markers such as Interleukin-6, C-reactive protein (CRP), and tumor necrosis factor-a (TNF-a) [24]. The involvement of RAAS indicates the role that hypertension, as one of the ORIs, plays in provoking AF.

Recently, the role of EAT in provoking LA remodeling has been under more research. For the sake of discussion, remodeling can be classified as either electrical or structural. Structural remodeling can be achieved through many factors; most importantly the stress obesity places on the human body with the added deposition of tissue which requires more blood volume which in turn places more stress on the cardiovascular system, mainly the left ventricle. This can lead to an increase in afterload leading to an increase in LA pressure and volume, and that in turn is associated with a left atrial enlargement (LAE) and distortions in LA dimensions in general, as discussed before. EAT might add to the process by provoking the fibrosis, characterized by the deposition of collagenous tissue in the myocardium mediated by myofibroblast, of adjacent atrial myocardium via secreting profibrotic factors including adipo-fibrokines [25]. It might be intuitive to think that the obese population has higher volumes of EAT, making them more susceptible to develop AF. This was revealed by a recent study in 2018 that found a significant association between obesity and volumes of EAT and also demonstrated by scanning for areas of low voltage, slow conduction, and voltage heterogeneity, that such changes are more pronounced in areas closer to EAT denoting the role of EAT in provoking AF [26], which in turn brings us to the point of electrical remodeling.

Electrical remodeling can be described as changes in the excitability of the cardiac myocytes and thus facilitating cardiac arrhythmias [27]. Such changes can affect ion channels and alter cardiac action potential as well as cardiac conduction, leading to slowing in conduction velocity and reduced action potential duration and eventually providing AF substrate [25,26]. In an interesting study, Erdem et al. investigated the association between BMI and electromechanical intervals in the atria in obese vs. non-obese populations. Data shows that LA diameter was higher in obese individuals, adding up to the evidence we showed earlier concerning LA dimensions, and also showing higher electromechanical delay both left intra-atrial and inter-atrial in the obese population suggesting that such findings can be an early subtle sign of arrhythmia in obese patients [28].

Lim et al. recruited 90 patients for noninvasive mapping of AF drivers as well as invasively assessing the degree of fractionation and endocardial voltage. Recruited patients were divided into two groups: 30 patients with persistent AF from the onset and 60 control individuals. Results demonstrated a significantly shorter baseline AF cycle length in patients with persistent AF from the onset compared to the control group. Additionally, patients with persistent AF from the onset showed lower endocardial voltage than the control group in both atria [15]. 

Another study investigated whether the electromechanical delay is prolonged in obese patients measured by tissue doppler imaging (TDI). The results show that obese patients had significantly higher intra- and inter-atrial electromechanical delays than normal-weight controls. The results also showed a correlation between the interatrial electromechanical delay and highly sensitive CRP levels (hsCRP), which is known to be associated with the presence of heart diseases [29]. This accumulating data showing the association between inflammatory markers and AF signifies the role of inflammation in the pathogenesis of AF and thus potentially the role it might play in preventing or reversing AF. The seemingly continuous process of subclinical inflammation of the atrial myocardium can explain the findings of incident AF in obese patients provoked by inflammatory markers, which are common operative pathophysiological pathways that can be present in one or all of ORIs. 

**Figure 1:**
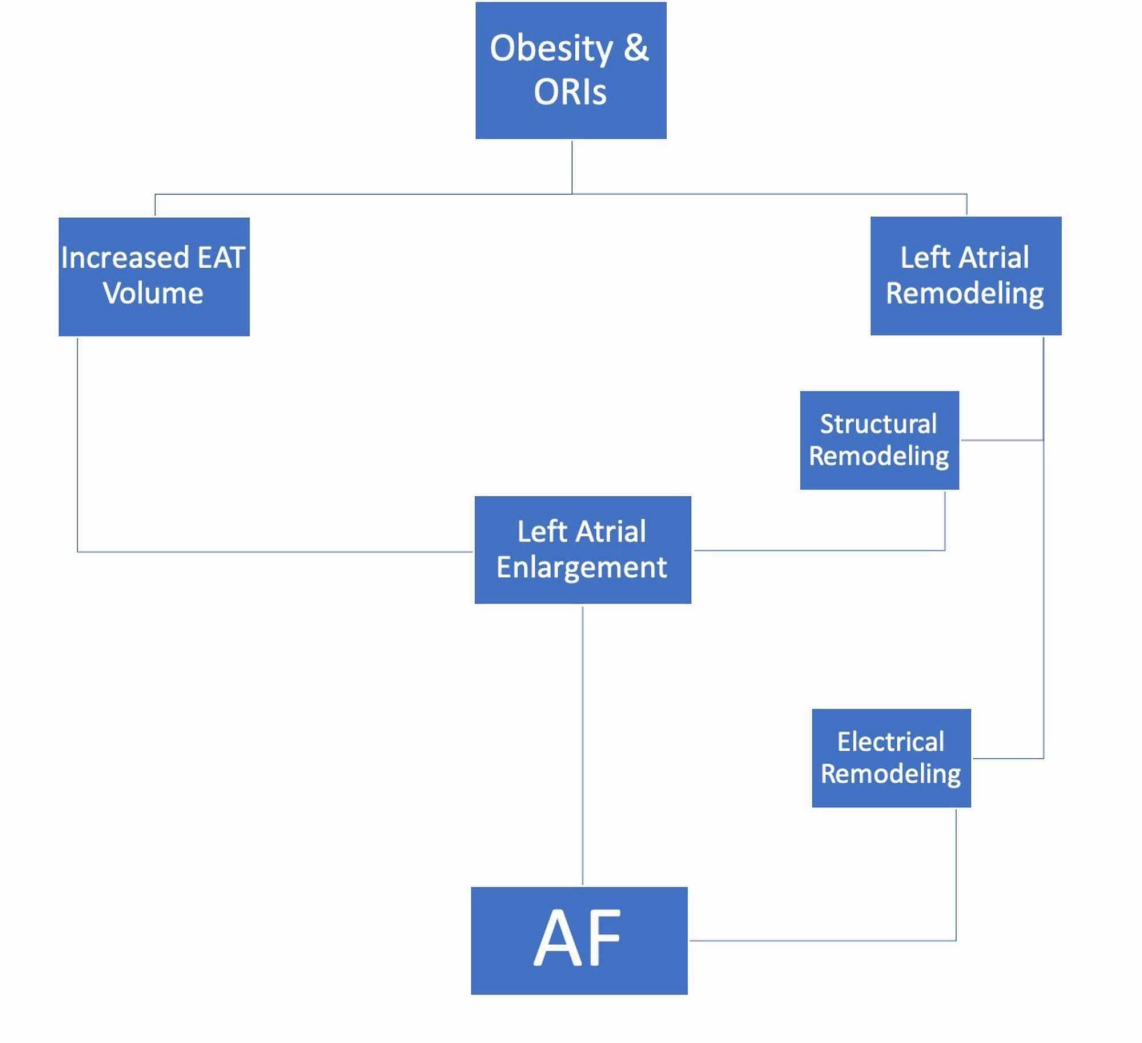
Pathological aspects of AF in obese patients ORIs = Obesity-Related Illnesses, EAT = Epicardial Adipose Tissue, AF = Atrial Fibrillation

Munger et al. exhibited that electroanatomical and electromechanical distortions can enable AF in obese patients. The latter had a shorter effective refractory period (ERP) in the left atrium, proximal, and distal pulmonary veins compared to the normal BMI population. Notably, mean LA pressure and LA volume index were higher in obese individuals compared to non-obese individuals [30]. Such mechanical and hemodynamic features of AF in obese population gives us a general idea of the massive stress obesity puts on the atria leading to the synthesis of AF substrate which can, in turn, provoke incidental AF, either in the form of paroxysmal AF or permanent AF and can as well help the progression of the underlying pathological process in the case of lack of detection and intervention. Please refer to Figure 1.

Potential approaches to management and treatment

Innovative approaches to detection and intervention are needed. Screening for AF in the general population appears to be highly controversial. We think it is best to detect AF, especially in the obese population, as early as possible for the sake of prevention hence the importance of the development of a screening tool that is highly sensitive, supported by evidence, and cost-effective. The European Society of Cardiology (ESC) recommends screening for AF in patients >65 years old either by short-term electrocardiogram (ECG) or pulse palpation [31]. At the same time, US guidelines lack any recommendations regarding screening for AF [32].

The cost and time needed for ECG examination could be a significant obstacle to a proper screening of AF. Thus, we propose selective screening as a potential early detection approach. Selective screening from our perspective should be applied to patients starting at a younger age than the one recommended by the ESC, hypothetically ≥55 years old, and more selectively towards obese patients (≥30 kg/m2) using pulse palpation or short-term ECG. The cut-off age proposed is based on the younger age range of obese patients who appeared to be more liable to AF according to the data collected, however, more research is required to determine a cut-off age based on evidence-likewise, a cut-off BMI.

Another approach for screening is technology. Technological advances have been opening new horizons in the medical field. The market of wearable technologies has been expanding rapidly with many devices that received and been seeking approval of the US Food and Drug Administration (FDA) to produce health data comparable to the concept of ambulatory ECG. Such technology is deemed to spark an evolution in healthcare, especially on the level of data collection and early detection. However, it carries with it a risk of over-detection of clinically irrelevant data or under-detection of clinically relevant data. Multiple types of wearable devices have been introduced into the market, including wrist devices and chest-strap devices. 

Halcox et al. conducted a randomized clinical trial (RCT) study called Assessment of Remote Heart Rhythm Sampling Using the AliveCor Heart Monitor to Screen for Atrial Fibrillation (The REHEARSE-AF study). The study included 1001 patients who are ≥65 years old with a CHA_2_DS_2_-VASc score of ≥2 and without AF; CHA_2_DS_2_-VASc is a scoring system used to estimate the risk of stroke in patients with non-valvular AF and stands for Congestive heart failure, Hypertension, Age ≥ 75 year, Diabetes history, Stroke history, Vascular disease, Age 65-74 years and Sex category where every category of these is worth one point if present except for two categories, age ≥ 75 years and stroke history, which would be worth two points if present. Patients were randomized to two arms; the first was an AliveCor Kardia monitor connected to a WiFi-enabled iPod to obtain ECGs (iECG) arm, and the second was routine care arm. Patients were followed for one year and results showed 19 patients diagnosed with AF in the iECG arm compared to five patients in the routine care arm (hazard ratio [HR], 3.9; 95% CI=1.4-10.4; P=0.007). Notably, patients in the iECG arm expressed the convenience such a technology provided as well as the peace of mind it delivered compared to conventional methods, although it is worth mentioning that the study reported a considerably high cost per one AF diagnosis in the iECG arm of $10,780 [33]. The AliveCor Kardia monitor is a smart-phone-compatible accessory that has two electrodes. The ECG is instantly shown on the smartphone screen after the placement of fingers on the electrodes. An automated algorithm on the mobile app checks the rhythm and it delivers a diagnosis through either of the following notifications “no abnormalities detected”, “possible atrial fibrillation” or “this ECG could not be interpreted”. The algorithm used by the mobile app has 97% sensitivity and 98% specificity for AF detection and is approved by FDA. Similar trials have been held with much smaller sample sizes using the same concept but maybe different devices. RCTs with bigger sample sizes will help us discover more feasible, economical, and reliable technology to detect asymptomatic AF in the obese population.

Multiple studies explored the conventional side of prevention and management. Abed et al., in their randomized clinical trial of 150 patients, demonstrated results of reduction of weight, AF symptom burden scores, and symptom severity scores in the intervention group compared to the control group. Their proposed intervention was weight reduction and intense management of risk factors. Further follow-up showed a reduction of interventricular septal thickness as well as decreased LA areas in the intervention group vs. the control group [34]. Such intervention seems logical and promising, but it’s worth noting that it could be unattainable at some levels when it comes to the general population. 

In a recent study, Tao et al. concluded the presence of a negative correlation between serum omentin-1 and the presence of AF and atrial remodeling. Serum omentin-1 is one of the adipokines known to be present in obesity, DM, and CAD. Further investigations under the same study showed that patients with permanent AF have even lower concentrations of serum omentin-1 than patients with paroxysmal AF [35].

Adiponectin is a copious adipose tissue-specific factor that seems to help increase insulin sensitivity as well as inhibit vascular inflammation [36,37]. It is largely secreted by adipocytes and can also be produced by cardiomyocytes, where it induces glucose and fatty acid uptake and 5′ adenosine monophosphate-activated protein kinase phosphorylation [38]. A study by Ybarra et al. investigated the relationship between adiponectin and LA size in uncomplicated obese patients. The study included 70 patients in which adiponectin levels were lower in the obese group compared to the non-obese groups. Adiponectin levels were demonstrated to be significantly lower in patients who are obese and have enlarged LA and showed a negative correlation with LA size even after controlling for age, sex, left ventricular mass, and Homeostatic Model Assessment of Insulin Resistance (HOMA-IR) and thus concluding that adiponectin level is a predictor of LA size [39].

Adiponectin appears to protect against the development of systolic dysfunction after myocardial infarction by facilitating the suppression of cardiac hypertrophy and interstitial fibrosis [40]. An RCT by Kourliouros et al. that included 90 consecutive patients who had cardiac surgery, and where 40% of the patients included manifested postoperative AF (36 patients), showed results of the lower release of epicardial adiponectin in patients with postoperative AF compared to patients who stayed in sinus rhythm postoperatively. Multivariate logistic regression analysis of risk factors for AF including epicardial adiponectin as an independent variable shown older age and epicardial adiponectin levels as independent predictors of postoperative AF suggesting the important role adiponectin plays as an anti-inflammatory mediator in such patients [41]. We think that serum omentin-1 and adiponectin show potential serum or laboratory markers to detect AF in obese patients. Such methods can be of great economic and timely value considering the cost and time that routine care could consume in case it was held as a means of screening. Additionally, adiponectin might have therapeutic benefits if we were able to develop it into a supplement for AF patients.

In line with the concept of intensive risk factor management, Soucek et al. conducted an RCT investigating the effect of short-term statin therapy on EAT volume, assessed by cardiac computed tomography in patients undergoing pulmonary vein isolation for treatment of AF. The study included 79 patients, of whom 38 received atorvastatin 80 mg/day, and 41 received placebo over three months. Results have shown that patients in the atorvastatin group exhibited a reduction in median EAT volume after three months of therapy (delta −4.6 (−8.9 to 1.3) cm3, P<0.05) along with a significant decrease in inflammatory markers as CRP and lipid profile levels. It is worth mentioning that the data of the study shows a larger baseline EAT volume in patients with persistent AF compared to patients with paroxysmal AF (100.4 (75.1-131.3) vs. 81.8 (57-108.2) cm3, P<0.05) [42]. In another study on DahlS.Z-*Lepr^fa^/Lepr^fa^* (DS/obese) rats, which was previously established as a model for metabolic syndrome, Yamada et al. treated the rats with atorvastatin in two groups, low dose vs. high dose. The high-dose group exhibited reduced adipocyte hypertrophy more than the low-dose group. Atorvastatin also showed that it could improve fibrosis and inflammation in the cardiac tissue, as well as adipose tissue inflammation [43].

Given the evidence-based role of EAT in AF in obese patients, measurement of EAT volume by an imaging modality can be of great value as a screening tool. Still, one limitation could be the high cost of imaging technique capable of detecting EAT volume, hence more research is required in such area.

Limitations

A limitation of our article was the scarcity of RCTs. Within the selected data, there were only three RCTs. The lack of RCTs could be justified by the focus of the research community on observational studies to acquire more understanding of the underlying pathological mechanism. Moreover, our research did not include pediatric patients with obesity.

## Conclusions

The complications of obesity are numerous and adds costly expenses to the healthcare system that is already encumbered. The complications of AF carry the same characteristics, and interestingly, both of them are connected and feed into each other. The prevalence of AF in obese patients appears to be alarming. The pathophysiological processes behind it are being explored quite vigorously, and we think that increased LA dimensions, as well as LA remodeling, are the two main processes driving it accompanied by the role of EAT in provoking the inflammatory and fibrotic elements of it. Based on the review it appears that the importance of high clinical suspicion of AF in obese individuals cannot be stressed enough. Prevention is the new medicine, and we can break the bond between obesity and AF by developing a preventive screening practice for AF in the obese population. The proposed selective screening shall be applied by pulse palpation or short-strip ECG starting at middle-aged obese individuals. More research is required on the regression of AF in patients who went under aggressive obesity management approaches as in bariatric surgeries. The role of imaging in detecting EAT volume and its relation to the prediction of AF in obese patients is a promising prospect. Lastly and most importantly, the development of a comprehensive protocol to detect and manage AF in obese patients is thought to be a necessity and of great value.
